# Beyond the Canon: Within-Plant and Population-Level Heterogeneity in Jasmonate Signaling Engaged by Plant-Insect Interactions

**DOI:** 10.3390/plants5010014

**Published:** 2016-03-16

**Authors:** Dapeng Li, Ian T. Baldwin, Emmanuel Gaquerel

**Affiliations:** 1 Department of Molecular Ecology, Max Planck Institute for Chemical Ecology, Jena 07745, Germany; dli@ice.mpg.de (D.L.); baldwin@ice.mpg.de (I.T.B.); 2 Centre for Organismal Studies, University of Heidelberg, Im Neuenheimer Feld 360, Heidelberg 69120, Germany

**Keywords:** jasmonate, plant-insect interaction, natural variation, hormone crosstalks, secondary metabolism, metabolomics

## Abstract

Plants have evolved sophisticated communication and defense systems with which they interact with insects. Jasmonates are synthesized from the oxylipin pathway and act as pivotal cellular orchestrators of many of the metabolic and physiological processes that mediate these interactions. Many of these jasmonate-dependent responses are tissue-specific and translate from modulations of the canonical jasmonate signaling pathway. Here we provide a short overview of within-plant heterogeneities in jasmonate signaling and dependent responses in the context of plant-insect interactions as illuminated by examples from recent work with the ecological model, *Nicotiana attenuata*. We then discuss means of manipulating jasmonate signaling by creating tissue-specific jasmonate sinks, and the micrografting of different transgenic plants. The metabolic phenotyping of these manipulations provides an integrative understanding of the functional significance of deviations from the canonical model of this hormonal pathway. Additionally, natural variation in jasmonate biosynthesis and signaling both among and within species can explain polymorphisms in resistance to insects in nature. In this respect, insect-guided explorations of population-level variations in jasmonate metabolism have revealed more complexity than previously realized and we discuss how different “omic” techniques can be used to exploit the natural variation that occurs in this important signaling pathway.

## 1. Introduction

Regulation of plants’ defenses during biotic stresses is a complex phenomenon. In nature, plants are essential nutritional sources for different communities of phytophagous insects. The selection pressure imposed by insect herbivory has likely shaped many of the strategies that plants employ to maximize their fitness in their native habitats. In addition to physical barriers (prickles, thorns or sticky trichomes) and constitutive chemical defenses that repel herbivores, plants also rely on more complex defense systems that are specifically produced during insect feeding [1]. These inducible anti-herbivore strategies involving multiple signaling and metabolic layers change a plant’s phenotype in order to antagonize insects’ growth and/or favor the recruitment of herbivores’ natural enemies. Many host plants of chewing insects have evolved the capacity to perceive elicitors in the insect’s oral secretions (OS) which triggers signaling pathways and ultimately the synthesis of a repertoire of defensive proteins and secondary metabolites [2,3,4,5,6,7,8]. In the case of highly-adapted insects that evolved counter-adaptations to some of these defensive compounds, the impact of these defenses is frequently limited to the slowing of insect growth or deterrence. As a direct or indirect result, it forces the herbivore to invest in detoxification processes, rather than outright toxicity [9,10,11]. As a consequence, plants also employ indirect defenses that increase the foraging efficiency of higher trophic levels that prey upon herbivorous insects by, for example releasing herbivory-induced plant volatiles (HIPVs) [9,12,13,14] or activating tolerance responses involving carbon or nutrient reallocation to less vulnerable plant parts, such as roots [15,16].

Most of the above described anti-herbivore direct and indirect responses are modulated across plant tissues and intrinsically connected with rapid changes in jasmonate pools [17]. Jasmonate biosynthesis and perception have been extensively reviewed [18,19,20,21,22,23,24] and are therefore not central to this review. Briefly, jasmonate acid (JA) and its derivatives are synthesized from linolenic acid (18:3) molecules released from chloroplast membranes galactolipids and via a first oxidative reaction catalyzed by lipoxygenase (LOX) proteins. (+)-7-iso-jasmonoyl-L-isoleucine (JA-Ile), the most bioactive jasmonate, has well-established roles in orchestrating many of the physiological processes important to plant-insect interactions [18,25,26,27,28,29,30]. The perception of jasmonate signaling necessitates the molecular action of the ubiquitin ligase SCF^COI1^ complex via a mechanism in which the F-box protein *COI1 (CORONATINE-INSENSITIVE1)* binds to JA-Ile and recruits *JASMONATE ZIM DOMAIN* proteins (*JAZs*) which are then targeted for ubiquitination and subsequent proteosomal degradation, leading to a release of *JAZ*-mediated repression of gene expression [31,32,33].

A first dimension of the specialization of the canonical jasmonate signaling lies in the tissue-specific modulation of jasmonates. Other dimensions of variations from the canonical pathway may result from signaling functions of jasmonates other than JA-Ile. 12-oxophytodienoic acid (OPDA) has notably been shown to activate certain physiological responses, such as tendril coiling and carnivorous trap functions and other transcriptional responses in a *COI1*-independent manner [34,35]. The fact that *Arabidopsis* possess 12 *JAZ* genes, some of which are subjected to alternative splicing, provides the basis for diversity in small molecule recognition and signaling that may be responsible for additional heterogeneity in jasmonate signaling amongst tissues [36,37]. An additional dimension in the jasmonate signaling variability space involves the analysis of natural variations existing between geographically distant populations and even in some cases among individuals from populations. In *N. attenuata*, herbivory-induced jasmonate levels greatly vary across geographically-distant accessions and this variation can partly explain differences in levels of secondary metabolite responses [38,39,40,41]. In a recent study, we explored the relationships among natural variation in jasmonate signaling and secondary metabolism to gain structural insights into previously unknown jasmonate-dependent secondary metabolites [40]. With the rapid advances in untargeted metabolome analysis, the combination of natural variation with deep metabolomic profiling and compound structural analysis, represent a very promising means of revealing the diversity in jasmonate signaling and the specialized metabolism that it regulates.

In this review, we describe modulations of jasmonate signaling in light of plant-insect interactions as illuminated by examples from recent work with the ecological model, *N. attenuata*. To this end, we provide a three-dimensional roadmap traversing within-plant, population-level to metabolic pathway-specific variations in jasmonate signaling and possible deviations from the canonical perspective. We also discuss means of manipulating jasmonate signaling by creating jasmonate sinks, micrografting procedures and a dexamethasone (DEX) system to disentangle tissue- and age-specificities in jasmonate signaling and their responses. Finally, we discuss the prospect of identifying novel jasmonate-regulated defense metabolites using natural variation in jasmonate signaling. In each of these sections, we highlight how deviations in jasmonate signaling can enhance our understanding of the phenotypic adjustments that are regulated during herbivory by this signaling pathway.

## 2. Within-Plant Heterogeneity in Jasmonate Signaling and Function

Tissue specificity in metabolism in response to herbivory is thought to be mediated, in part, by tissue-specific deviations in jasmonate signaling [42]. Spatial variations in the expression of the family of *JAZ* genes suggests that the diversity is not simply an artifact of gene redundancy. Despite the current consensus that *JAZ* proteins act as general repressors of jasmonate signaling, little is known about how individual *JAZ* proteins function. Even in the model plant *Arabidopsis*, phenotypes of plants with individually silenced *JAZ* proteins remain incomplete, and their tissue-specific functions remain poorly understood [43,44]. *JAZ* genes are differentially expressed in locally elicited leaves, systemic unelicited leaves and roots of *N. attenuata* when elicited by a standardized method mimicking *Manduca sexta* herbivory (wounding plus application of herbivore oral secretions (W+OS)) [37]. However, this tissue-specific *JAZ* expression is not fully understood and additional work is needed to spatially dissect the function of this *JAZ* regulatory network. Recently, *JAZ10* was successfully employed as a JA-responsive reporter in *Arabidopsis* to identify root-specific activators or repressors of wound signaling [45].

**Figure 1:**
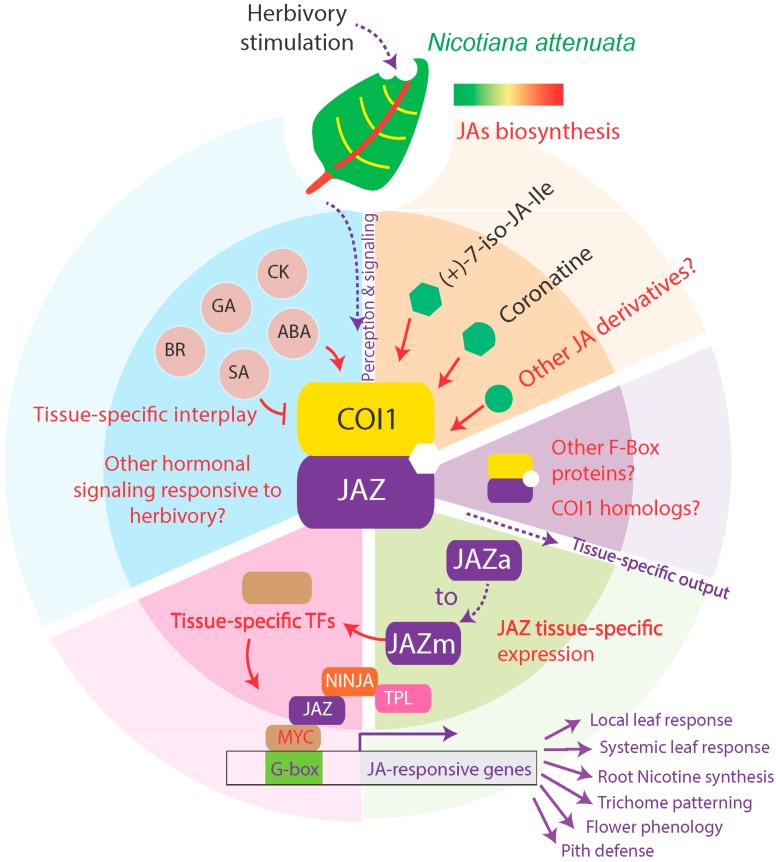
Schematic model of modulations in jasmonate perception, signaling and corresponding tissue-specific herbivory-elicited responses. New discoveries in jasmonate signaling in the ecological model, *Nicotiana attenuata* are presented. The small circle at the top represents jasmonate biosynthesis after herbivore attack; the inner circle represents jasmonate perception and signaling, the outer larger circle represents outputs of tissue-specific responses. The six different types of modulations from the canonical model of jasmonate signaling are presented in different colors. White, jasmonate biosynthesis deviates in different tissue types of leaves; orange, other jasmonate derivatives that are likely to serve as ligands need to be discovered; purple, other F-box proteins may serve as functional groups; green, tissue-specific *JAZ* expressions need to be fully investigated; pink, tissue-specific transcription factors are yet to be discovered; blue, deviations of hormonal crosstalk in different tissues. Red texts indicate areas where modulations are likely to take place. *TPL, TOPLESS.*

Interactions with other hormonal signaling are also likely to contribute to these tissue-specific responses. For example, specific sets of genes encoding auxin- and ethylene-related signaling processes show OS-elicited responses in systemic leaf tissues that are distinct from those in locally treated tissues [46]. In *Nicotiana tabacum*, jasmonates regulate the expression of *NtPYL4* which encodes an ABA receptor protein affecting the regulation of defensive alkaloids biosynthesis in roots [47]. An antagonistic crosstalk among brassinosteroids (BRs) and jasmonates specifically alters trichome density and defensive metabolite accumulation in tomato [48]. Gibberellins (GAs) interplay with jasmonate signaling through the competitive binding of DELLAs, GA signaling transcriptional repressors, to *JAZ* proteins [49]. The balance of the two negative regulators is flexible according to tissue type [50]. In contrast to the GA-JA synergism in stamen filament development and antagonism in aboveground defense-growth tradeoffs [51,52], a crosstalk between GA and JA has not been yet reported in roots. The interplay between salicylic acid (SA) and jasmonates depends on their tissue-specific concentrations, as SA and JA act synergistically at low concentrations but antagonistically at high concentrations [53]. The kinetics of hormone accumulation after elicitation strongly influences hormonal crosstalk. When SA and JA pathways are activated simultaneously, SA antagonizes JA signaling; however this repression is largely abolished when the two signaling pathways are not synchronously activated [54]. Jasmonate-based defense responses to insect herbivory not only vary across tissues but also throughout the ontogeny of a given tissue as a result of complex rearrangements of these interactions among hormonal pathways [42]. The influence of ontogeny becomes apparent when comparing young with old or even senescing leaves with respect to the amplitude of their herbivory-induced jasmonate bursts and the defense responses they activate. Herbivory-induced jasmonate bursts have been repeatedly shown to be consistently lower in older than in younger tissues. Cytokinins are well known for their roles in inhibiting leaf senescence, a process which jasmonates counter [55,56]; this interplay between the induction of cytokinins and jasmonates in response to herbivory has been shown in both local and systemic tissues [57]. Clearly powerful phenotyping tools combined with tissue- and age-specific genetic manipulations are required to fully address how jasmonate-signaling changes over ontogeny and senescence. A schematic model of jasmonate signaling and responses to insect herbivory in *N. attenuata* is presented in Figure 1.

### 2.1. Jasmonate Signaling within-and in Between-Leaves

When chewing herbivores attack a leaf, a short-distance mobile signal is rapidly triggered, which in turn produces a long-distance mobile signal that moves to unattacked leaves following vascular connections. This short-distance mobile signal travels in particular directions towards the undamaged parts of the attacked leaf where it triggers region-specific jasmonate signaling, while the long-distance signal propagates between leaves to elicit jasmonate signaling and defense responses in distal leaves. Relatively little is known about which signal serves as the initial trigger and how the signaling crosses from wounded leaves to other plant parts. Electric and hydraulic signals are thought to play a central role in this process [58,59,60]. Recently, a major advance in understanding the mechanisms involved in the electrical component of the signal was achieved when *GLUTAMATE RECEPTOR-LIKE* genes (*GLRs*) were found to be involved in the propagation of electrical signals and to elicit wound-induced expression of several *JAZ* genes [61].

The importance of jasmonate signaling for systemic defensive traits regulation during insect herbivory was first revealed by the seminal work of C.A. Ryan on the induction of digestive proteinase inhibitors in the *Solanum Lycopersicum* tomato [26,27]. Several decades of research from multiple laboratories on the genetic bases of jasmonate metabolism and on the regulation of defense metabolism in insects have firmly established the pivotal role played by jasmonates in orchestrating many of the defenses activated at the whole plant-level that antagonize insect performance [62]. During insect feeding, jasmonate signaling is repeatedly remodeled in both local and systemic tissues by herbivore and damage associated molecular pattern (HAMP and DAMP) elicitors. Jasmonate dynamics detected within- and between-leaf are qualitatively and quantitatively different. In locally-attacked leaves, HAMPs and DAMPs reconfigure the wound response and amplify jasmonate biosynthesis. Local herbivory-induced jasmonate dynamics can be variable across natural populations as well as closely-related species as a result of variations in a plant’s ability to perceive HAMP and DAMP elicitors [63,64]. Importantly, rapid changes in jasmonate dynamics occur not only in herbivore-damaged leaves but also in intact systemic tissues [28]. In systemic leaves, jasmonates are synthesized *de novo* in response to transported signal activated by HAMP and DAMP elicitors from damaged leaves [65]. The long distance signaling systems operate at different temporal and spatial scales from those of the short-distance systems. The amount of the jasmonate accumulation and the persistence of the jasmonate levels in undamaged systemic leaves differ from those in wounded leaves [65].

In many species, compositional variations in jasmonate profiles have been observed both within and between leaves. In *Arabidopsis*, jasmonate metabolic profiles differ dramatically both in their compositions and accumulations in systemic leaves and midveins of systemic leaves compared with those in local leaves in response to wounding; while within the wounded leaf, certain jasmonate derivatives specifically accumulate in the midvein, distinguishing the jasmonate signatures from those in the lamina [66]. In *N. attenuata*, the conversion efficiency from JA to JA-Ile greatly differs for different positions relative to the wound site [17]. Constraints in the spread of herbivory-elicited *MAPKs* (*mitogen-activated protein kinases*) activation are responsible for the inhomogeneity of the jasmonate accumulation and transcriptional responses in different areas of the leaves following simulated insect herbivory [67]. Most jasmonate biosynthesis enzymes are present at high levels in vascular bundles [68,69] reinforcing the pivotal roles of midveins and leaf secondary veins in shaping the spatial heterogeneity of jasmonates within a leaf [17]. Along this line, *Arabidopsis* *LOX6*, a lipoxygenase responsible for rapid jasmonate synthesis in tissues distal to wounds, has been shown to be expressed in a group of cells that are tightly associated with xylem vessels. These regions where contact cells are located are highly sensitive to the release of water column tension in response to wounding. The squeeze cell hypothesis elaborated by E.E. Farmer suggests that contact cells that are hydrostatically coupled to the xylem are likely to be squeezed by pressure increases in the xylem after wounding which disperses hydrostatic pressure finally triggers jasmonate synthesis in distal tissues [70]. Although it is clearly recognized that vascular connectivity among leaves greatly conditions the amplitude of distal jasmonate bursts and dependent-responses, the role of secondary veins in organizing within-leaf spatial spread of jasmonate signaling will requires additional study.

### 2.2. Root-Mediated Jasmonate Signaling

Root growth inhibition was one of the first observations of a plant’s physiological responses to jasmonates and has been used to screen jasmonate-deficiency and insensitivity which led to the discovery of many important signaling components of the jasmonate pathway such as *JAR1, COI1, JIN4, JIN1/MYC2, JAI3* or *AXR1*. Root-specific molecular aspects of the jasmonate pathway have been recently investigated in depth with the discovery that the organizational principles of the jasmonate signaling machinery differ significantly between the root and the shoot. In this respect, *NINJA* plays a critical role in repressing jasmonate signaling in roots and thereby maintaining the homeostasis of root growth [45]. A follow-up study provided experimental support for a negative regulatory role of *MYC2* together with *NINJA* in constraining jasmonate signaling during normal root cell division and elongation after wounding [71]. These studies provide a molecular framework for understanding the differences in jasmonate signaling between above and belowground organs. In these studies, a *JAZ10* reporter was used to screen for mutants affected in the organ-specific activation of jasmonate signaling in *A. thaliana* seedlings, and highlights the need for tissue-specific manipulations to finely dissect the complexity of jasmonate/*JAZ* regulatory networks.

Relatively little work has been conducted on the role of jasmonate signaling in the regulation of defensive metabolism in roots. The systemic responses in roots during leaf herbivory can exceed the amplitude of some of the best systemic responses detected in aboveground tissues. For instance, the transcriptomic changes activated in roots after *Spodoptera littoralis* herbivory of maize leaves exceeded those observed in damaged leaves [72]. Consistent with this, genes and metabolites that are differentially perturbed between locally damaged and systemically intact tissues in response to *M. sexta* leaf herbivory in *N. attenuata* exhibit more pronounced differential responses in roots than in the shoot, suggesting a root-specific contribution to leaf-based immunity [46]. Jasmonate signaling and dependent responses are not only tissue-specific but also dependent on the systemic organs in which elicited responses take place. The jasmonate metabolic profiles of systemic leaves of root-induced plants varies significantly from the profiles of systemic leaves of shoot-induced plants indicating that roots and shoots likely possess different jasmonate signaling pathways that allow plants to tailor their responses optimally to the organs that are damaged [73,74].

When leaves of tobacco plants are mechanically wounded or attacked by a shoot herbivore, the plants activate synthesis of nicotine in the roots which is then mobilized to the shoot [4]. In *N. attenuata*, a long-distance signal travels from damaged leaves to roots and initiates the transcriptional up-regulation of nicotine biosynthetic genes in the roots [75]. Surprisingly, impairment of jasmonate synthesis and perception in undamaged roots is associated with a priming of jasmonates and abscisic acid (ABA) levels in damaged leaves in response to wounding, indicating that a shoot-root-shoot signaling loop coordinates this whole-plant signaling system [76].This result highlights the important point that to comprehensively elucidate patterns of heterogeneity in jasmonate signaling, an understanding of the integrating signaling networks created by the interactions of multiple hormonal pathways is required.

Many plants exhibit an increase in carbon (C) transport from locally damaged leaves as well undamaged systemic leaves to roots in response to herbivore elicitation [16,77,78,79,80,81,82]. Such C reallocation might, on the one hand, result in herbivory-induced tolerance via increased C storage and on the other hand, buffer against carbohydrate depletion in roots. Simulated M. sexta herbivory reduces starch and sugar contents in roots of *N. attanuata* which may enable plants to support the synthesis of plant defensive metabolites belowground to attain full defense deployment. Jasmonate signaling plays a key, but not exclusive, function in the regulation of shoot and root carbohydrate turnover.

### 2.3. Jasmonate Signaling in Tissues Other Than Leaves

Insect herbivory to plant stems results in distinct jasmonate patterns compared to roots and leaves. When *N. attenuata* is attacked by the larvae of the pith-feeding weevil *Trichobaris mucorea*, it activates a separate spatial defensive strategy in which JA and JA-Ile levels specifically increase in the damaged pith region but not in connected leaves (manuscript in preparation) [83]. Another interesting example of tissue-specific modulation of jasmonate signaling during plant-insect interactions concerns the dramatic shifts in flower phenology in *N. attenuata*, a phenomenon which is elicited by *M. sexta* leaf herbivory and requires jasmonate signaling. The larvae of *M. sexta*, the important night pollinator hawkmoth of *N. attenuata* flowers, are important herbivores and nectaring during pollination increases oviposition rates. This creates a dilemma for *N. attenuata* in how to attract pollinators without increasing herbivory rates. When *N. attenuata* is attacked by *M. sexta* larvae, the phenology of flowers shifts from night-opening and benzyl acetone scenting flowers to morning-opening flowers that do not scent at night and these flowers attract day-active hummingbirds. This herbivory-elicited shift in floral phenology requires jasmonate signaling [84]. Although the underlying regulatory mechanism of the flower opening phenology remains unclear, jasmonates play a central role in floral development and maturation [85], and the speed and rate of corolla opening is highly dependent on jasmonate signaling [86].

Another tissue-specific jasmonate-dependent developmental response related to defense against insects is induced trichome formation. Trichomes often play a role in resistance due to the physical barrier they present to herbivores and for being sites of biosynthesis of various secondary metabolites such as terpenoids, flavonoids and alkaloids that function as direct defenses [87,88] as well as acyl sugars that function as indirect defenses by increasing the predation pressure on attacking herbivores [89]. The tomato mutant *odorless-2* shows altered morphology, density and metabolites in their glandular trichomes, and as a consequence is susceptible to increased attack from both *M. sexta* and Colorado potato beetle larvae in the field [90]. In *Arabidopsis*, endogenous jasmonates regulate trichome initiation via the *COI1* receptor complex to activate the trichome-specific transcription factors *MYB75* and *GLABRA 3* (*GL3*) leading to development of metabolically active trichomes [91,92]. Wound-induced trichome formation is mediated by canonical SCF^COI1^-dependent jasmonate signaling as *aos* and *coi1*, two mutants that completely lack endogenous jasmonate function show reduced trichome numbers after wounding. Intriguingly, the jar1 mutant which is deficient in the generation of the most bioactive jasmonate compound JA-Ile [93] is unaffected in trichome production. This observation suggests that although JA-Ile is the active ligand of SCF^COI1^, other active ligands may be responsible for trichome induction [94].

## 3. Manipulating Tissue-Specific Metabolic Sinks in Jasmonate Signaling

The most commonly used means of studying hormonal function involves, silencing or knockout mutations of key genes in hormonal biosynthesis pathways [95]. Substrate availability also severely determines jasmonate biosynthesis output and creating metabolic sinks has previously been shown to yield clean metabolic and signaling outputs by diverting substrates of the targeted metabolites [96,97]. Manipulating methylation, one of the catalytic reactions used by plants to adjust their pools of hormones, represents a promising approach to unravel jasmonate signaling output and its downstream homeostatic systems [98,99,100]. Ectopically expressing *AtJMT* gene in *N. attenuata* redirects jasmonate flux towards MeJA which competes with the production of the bioactive form JA-Ile and thereby down-regulates dependent defensive responses [101,102]. This redirection of jasmonate metabolism “silences” jasmonate signaling; the creation of metabolic sinks of jasmonates impairs the accumulation of defense-related transcripts but not transcripts of jasmonate biosynthesis genes and the upstream genes in the jasmonate pathway [101]. With these “jasmonate sink” transformed plants, redirection of jasmonate flux was more pronounced in the midveins and petioles compared with laminas and depleting JA levels clearly accentuated heterogeneities in the jasmonate signatures between local and systemic leaves elicited by simulated herbivory [101]. In *Arabidospsis*, overexpression of *AtJMT* results in elevated levels of MeJA and normal levels of JA but constitutively enhanced expression of JA biosynthesis related genes and plant resistance to stresses [103]. It is noteworthy that *N. attenuata* plants ectopically expressing *AtJMT* do not differ from wild type plants in morphology at the rosette stage of growth. When plants reach flowering stage, the overexpression of *JMT* in *N. attenuata* severely alters floral organ maturation, corolla limb opening and volatile emissions which is consistent with the key function of JA-Ile in regulating these processes [86]. Manipulating tissue-specific jasmonate sinks therefore represents a promising approach to understand the nature of jasmonate homeostasis systems in different organs.

Given the spatiotemporal specificity of jasmonate signaling and the similar specificity in the activity patterns of interacting organisms, it would be ideal to have sufficient control over gene expression to manipulate these dynamic interactions in real time in different tissues and ideally also under field conditions. [104,105,106] Dexamethasone (DEX) inducible control over gene expression was first applied in *Arabidopsis* to dissect systemic jasmonate signaling [65] and is one of the most sensitive inducible promoter systems available for plant research [107]. Recently, a DEX-inducible pOp6/LhGR expression system was developed for *N. attenuata* and applied to the spatiotemporal modulation of cytokinin signaling during interactions with native herbivore *Tupiocoris notatus* in both laboratory and field studies [65,108]. This DEX-inducible transactivating system contains two transcription units: The first unit employs a constitutive CaMV 35S promoter to express a DEX-responsive chimeric transcription factor (LhGR). The second unit consists of six copies of the transcription factor binding site (lac operators “pop”) upstream of a minimal CaMV 35S promotor controlling the expression of inverted repeats of the target gene. In this process, spatial resolution in gene silencing is achieved based on the tissue site of DEX application. As such, this DEX-inducible system allows for surgical manipulations of tissue-specific traits while minimizing off-target effects in other tissues, which may confound interactions with non-targeted organisms [108]. For instance, when inducing the growth of equal-sized lateral branches by apical meristem decapitation, it is possible to achieve branch-specific gene silencing manipulation by the application of the DEX treatment to specific branches without cross-silencing of adjacent branches [108]. This field-applicable DEX-inducible tool when combined with the *JMT/JME* jasmonate sink expression system in *N. attenuata* plants, will provide an ideal means of disentangling tissue-specific regulation of jasmonate signaling for some of the plant-insect interaction-dependent responses described above. A new frontier will consist in the combination of this DEX-transactivating system with tissue- or cell type-specific promoters when relevant ones are identified in *N. attenuata*.

Additionally, (micro)grafting techniques have provided significant insights into the study of systemic signaling. Grafting experiments with tomato mutants in jasmonate biosynthesis and signaling were first employed to reveal that local jasmonate signaling was required to mount systemic responses in distal leaves [109]. Recently, a simple and efficient micrografting method first developed for *Arabidopsis* has been developed to manipulate below- and above-ground parts in *N. attenuata* for the study of shoot-root signaling in plant-insect interactions [110]. The main limitation of this micrografting method is that specific gene silencing is restricted to the root due to the fact that RNAi silencing can move rootward from the shoot. Despite this limitation, the micrografting procedure represents an important advance when combined with silencing/overexpression of genes mediating jasmonate biosynthesis or perception in understanding root- and shoot-specific characterization of gene function [76]. For example, such micrografting combinations of *N. attenuata* lines have been used to reveal that the transportation of nicotine from roots to shoots requires jasmonate synthesis and JA-Ile perception in both shoot and root compartments [76]. Additionally, phytohormone profiling of these micrografting combinations revealed an unexpected elevation of ABA levels in damaged leaves of both micrografted empty vector (EV) (shoot)/RNAi COI1 (root) and EV/RNAi *AOC*. This result highlights the importance of the hormonal crosstalk between JA and ABA in the shoot-root-shoot signaling interplay established during leaf herbivory and induced nicotine mobilization to the shoot.

## 4. An insect-Guided Tour of Population-Level Polymorphisms in Jasmonate Signaling and Dependent Responses

Considerable amounts of natural variation in jasmonates and dependent responses have been identified in many species [39,41,111,112]. In *Arabidopsis*, MeJA induced gene expressions and glucosinolate accumulation are highly variable among different accessions [111,113]. Modulations of jasmonate accumulation capacities dramatically influence a plant’s performance in nature, notably by affecting the inducibility of insecticidal secondary metabolites. For herbivorous insects, the choice of host plants is essential for their survival and reproduction and insects have therefore evolved complex sensing and behavioral responses to physical and chemical characteristics of their host plants. In this section, we describe interrelationships between modulations of jasmonate signaling and defense metabolism responses and how these affect a plant’s interaction with insects as well as insects’ host choice. We also discuss how the survey of natural insect populations (“insect-guided tour”) can be used to identify natural variations in jasmonate accumulation and signaling [114].

*N. attenuata* populations exhibit a high genetic diversity within populations, which can be as great as that seen among populations [115] and this is thought to be in part due to the “fire-chasing” germination behavior of this annual plant in which seeds from long-lived seed banks synchronously geminate in response to smoke-derived germination cues [116,117]. This post-fire germination behavior could result in high within-population variation due to the recruitment of plants of very different ages into post-fire populations from the long-lived seed banks. Considerable natural variation in herbivory-induced jasmonate signaling and secondary metabolites have been reported from this species [38,39,40] and *N. attenuata* genotypes accumulating low amounts of jasmonate are more severely damaged by folivores such as grasshoppers, leafhoppers, lepidopteran larvae, beetles and stem-boring weevils in the field [83,118].

Some phytophagous insects appear to directly sense the jasmonate signaling and/or dependent responses of their host plants to evaluate their suitability as food. For example, the corn earworm *Helicoverpa zea* can respond to jasmonate signals to upregulate the expression of genes important in the detoxification of host plant defenses [119]. *Empoasca* leafhoppers have also evolved mechanisms to select host plants in nature by eavesdropping on their JA-mediated signaling capacities. This probe-feeding insect preferentially targets jasmonate deficient plants [39], but this choice preference is independent of major jasmonate-inducible defense metabolites as well as detectable changes in plant volatiles as revealed from the qualitative and quantitative evaluation of damages from the attack of *Empoasca* leafhoppers on untransformed and jasmonate signaling-deficient transgenic plants under field conditions and the measurement of jasmonate-regulated direct and indirect defense compounds. On the other hand, the special fire-synchronized germination behavior of *N. attenuata* exposes these ephemeral host plants to herbivores from various guilds that colonize the environment after fire, but also to an intense selection for rapid growth with conspecifics and intense competition for resources. Plant’s growth-defense trade-offs involving jasmonate regulation might account for the maintenance of low jasmonate-producing genotypes in native populations, providing them with competitive advantages, despite the clear disadvantage of being defenseless and frequently targeted [114].

Jasmonate signaling plays a decisive role in modulating regrowth responses after *M. sexta* elicitation among different natural *N. attenuata* populations and across populations, herbivory induced jasmonate production negatively correlates with flower production of regrowing shoots [120]. Recent studies have revealed additional complexity in the jasmonate signaling pathway and its correlations with the direct and indirect defenses that it regulates. Herbivory-induced concentrations of phytohormones differ significantly among *N. attenuata* accessions, and that individuals even within small populations emit significantly different HIPVs following simulated herbivore elicitation [38] which include trans-α-bergamotene, an abundantly produced herbivory-induced sesquiterpene which functions as an indirect defense in nature. Polymorphisms in endogenous jasmonate signaling among accessions induced by herbivory surprisingly barely correlate with variations in HIPVs profiles. Consistently, when comparing *N. attenuata* Arizona (AZ) and Utah (UT) accessions, the AZ accession was shown to exhibit similar capabilities in producing HIPVs despite having jasmonate accumulations that are half those observed in the UT accession [121,122].

## 5. Using Natural Variation to Bridge the Gap Between Jasmonate Signaling and Elicited Metabolites

**Figure 2:**
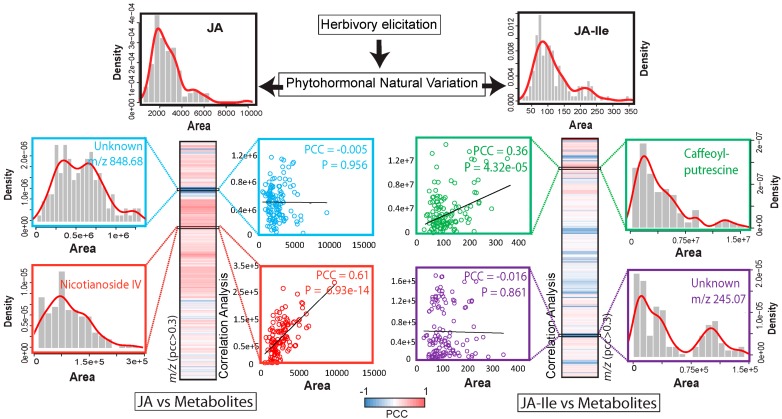
Population-level quantitative variations in herbivore-elicited metabolites only partly overlaps with jasmonate accumulation polymorphisms. The figure is modified from [40]. Density distribution plots of JA and JA-Ile (x axis, area of intensities and y axis, fitted density with histogram) (123 samples) illustrate the patterns of natural variation in JA and JA-Ile levels as analyzed by targeted LC-MS/MS/MS workflows for leaf samples collected 1 h after simulated herbivory from glasshouse-grown accessions of *N. attenuata*. Heatmaps of pairwise Pearson correlation coefficients (PCCs) (only PCCs with either JA or JA-Ile >0.3 are shown) illustrate significant co-regulation patterns between metabolite relative levels and JA and JA-Ile levels. Examples of known and unknown metabolites are depicted in density plots and scatter plots (colored with different color boxes accordingly). The herbivory-inducible defense compound, Nicotianoside IV, correlates significantly with JA whereas N-caffeoylputrescine shows significant correlation with JA-Ile. Unknown *m/z* 848.68 and 245.07 exhibit poor correlations with JA or JA-Ile. Discovering the identity of these and other unknown compounds exhibiting significant correlation scores with JA and JA-Ile levels will be the topic of future research to uncover novel defensive metabolites in *N. attenuata*.

Natural variation has been extensively used to discover the function of genes. With current advances in sequencing and with the use of multi-parent advanced generation inter-cross (MAGIC) and recombinant inbred lines (RILs) combined with analytical approaches such as liquid chromatography-mass spectrometry (LC-MS) and NMR for targeted metabolic analysis, the exploration of natural variations in both model and crop species can now be used to identify the genetic fundamentals of metabolic traits via quantitative traits locus mapping [123,124,125]. Large-scale metabolic quantitative trait loci (mQTL) combined with correlation-based network analysis can powerfully query the genomic regions harboring particular secondary metabolic traits [126,127]. Similar approaches can be used in the analysis of large-scale defense metabolism by correlating the variation across different genotypes with the aim of identifying naturally variable and jasmonate-dependent metabolic traits. To implement such an approach, the challenge of the large-scale acquisition of analytical data on as many secondary metabolites as possible for a given tissue needs to be addressed. Hypotheses about the identity of unknown metabolites can be evaluated by patterns of co-regulation for intermediates within a biosynthetic pathway [128]. Such approaches can be progressively expanded to other metabolites beyond those with already known pathway assignments by correlating their variation with those detected at the level of metabolic genes, such as recently implemented in rice and maize and but also for non-model system plants [129,130,131,132]. Recently, we developed a metabolomics and computational pipeline using a non-targeted tandem mass spectrometry (MS/MS) approach to resolve the chemodiversity existing in secondary metabolic responses to herbivory elicitation in native populations of *N. attenuata* [40]. The non-targeted MS/MS approach generates non-targeted MS/MS data for all mass signals detected and allows researchers to capture a comprehensive picture of the metabolic diversity detected in these natural populations. Moreover, the use of natural variation patterns across genotypes of a species for a given tissue (in this study, rosette leaves) can support the annotation of previously poorly explored metabolite groups and in theory be extended to other types of sample comparisons, for instance to monitor tissue-wide metabolic variations. Navigating the metabolic natural variation map created in this study showed that both jasmonate metabolism and plants’ defensive metabolites elicited by herbivory exhibit high diversity among accessions. However, investigating the correlation between these secondary metabolites induced by herbivory and induced jasmonate levels revealed that the patterns of natural variation detected for these metabolites only partly overlap with upstream variations in jasmonate production [40]. Significant correlations were detected for some of these herbivory-inducible metabolites with either JA or JA-Ile levels but rarely with both. This partial uncoupling between jasmonate variations and quantitative differences in many of the metabolic profiles after herbivory suggests that the jasmonate-mediated metabolic accumulation mechanism is under a complex regulation system which might involve additional components besides the canonical jasmonate signaling pathway (Figure 2).

Non-targeted MS/MS profiling has the potential to identify unknown metabolites that could be involved in anti-herbivore defenses. The problem is that stress-responsive secondary metabolites are extremely structurally diverse and most plant metabolite databases are too sparsely populated to provide useful hints on these unknown structures. Innovative approaches have therefore to be implemented to accelerate the annotation of MS/MS data. One of the successful approaches pioneered by the Dorrestein laboratory involves the creation of MS/MS molecular networks. This approach involves the generation of networks in which mass spectra (nodes) from one data-set are clustered using edges whose length is inversely proportional to the pairwise similarity between spectra. This molecular networking facilitates the mining of unknown metabolites clustered adjacent to known ones. The network view of large metabolomics data-sets also allows for the combined visualization of other biological information linked to a given metabolite (MS/MS spectrum). This possibility was used to visualize statistical descriptors of natural variation for each MS/MS spectrum (nodes in the network) [40], and co-regulation patterns with different jasmonates. More generally, we predicted that such MS/MS network approaches can profoundly advance our understanding of the metabolic heterogeneity associated with modulations of the jasmonate signaling pathway (Figure 2) among different tissue types and/or as result of different stress conditions.

## 6. Conclusions and Perspectives

More than 50 years of research in jasmonate metabolism and regulation have established the importance of this signaling pathway for a plant’s ecological interactions, particularly those with insects. An increasing number of studies demonstrate that tissue-specific responses to herbivory are also associated with tissue-specific regulation in jasmonate signaling. The development of more sensitive and comprehensive analytical tools becomes increasingly important to improve the analysis of temporal modulations across multiple tissues and of the metabolites that are regulated downstream and function as defenses during herbivory. The acquisition of MS/MS metabolite data and the generation of structurally rich MS/MS similarity networks will facilitate the exploration of the multiple metabolic layers that make up plants’ defense responses. The analysis of natural variation in metabolic responses harbored in native plant populations will greatly enrich our understanding of the diversity in metabolic outcomes that jasmonate signaling can trigger and provide a healthy antidote to the typological thinking that emerges from focusing too much on models raised in the rarefied environments of growth chambers with little of the environmental complexity that sculpted the diversity of specialized metabolism.
